# Clinical applications of artificial intelligence in identification and management of bacterial infection: Systematic review and meta-analysis

**DOI:** 10.1016/j.sjbs.2024.103934

**Published:** 2024-01-14

**Authors:** Mohammad Zubair

**Affiliations:** Department of Medical Microbiology, Faculty of Medicine, University of Tabuk, Tabuk 71491, Kingdom of Saudi Arabia

**Keywords:** Artificial Intelligence, Convolutional Neural Network, Neural Network Algorithms Pediatrics, Pneumonia

## Abstract

Pneumonia is declared a global emergency public health crisis in children less than five age and the geriatric population. Recent advancements in deep learning models could be utilized effectively for the timely and early diagnosis of pneumonia in immune-compromised patients to avoid complications. This systematic review and *meta*-analysis utilized PRISMA guidelines for the selection of ten articles included in this study. The literature search was done through electronic databases including PubMed, Scopus, and Google Scholar from 1st January 2016 till 1 July 2023. Overall studies included a total of 126,610 images and 1706 patients in this *meta*-analysis. At a 95% confidence interval, for pooled sensitivity was 0.90 (0.85–0.94) and I2 statistics 90.20 (88.56 – 91.92). The pooled specificity for deep learning models' diagnostic accuracy was 0.89 (0.86–––0.92) and I2 statistics 92.72 (91.50 – 94.83). I2 statistics showed low heterogeneity across studies highlighting consistent and reliable estimates, and instilling confidence in these findings for researchers and healthcare practitioners. The study highlighted the recent deep learning models single or in combination with high accuracy, sensitivity, and specificity to ensure reliable use for bacterial pneumonia identification and differentiate from other viral, fungal pneumonia in children and adults through chest x-rays and radiographs.

## 1 Introduction

Pneumonia is an acute lower respiratory tract infection that causes inflammation of alveoli, and air sacs due to pleural effusion and pus accumulation in one or both lungs resulting in hypoxemia as the strongest mortality predictor (Kundu et al., 2021). This communicable disease mainly occurs due to more than thirty causative factors, mainly grouped into bacterial, viral, mycoplasma, or fungal infection with a global incidence rate of 12 % (Yue et al., 2020, Pal and Das, 2023). The “Global Burden of Disease, Injury, and Risk Factor- GBD” study (2017) stated pneumonia is the leading cause of mortality among pediatrics less than five years old and a geriatric population greater than seventy-year age (Chumbita et al., 2020). 450 million people worldwide experience pneumonia each year which results in 4,000,000 deaths due to delayed diagnosis (Vidhya et al., 2022). UNICEF and World Health Organization (WHO) due to the high incidence of morbidity and mortality declared pneumonia as a chief humanitarian emergency by naming it a “children forgotten killer” in the “Global Action Plan for Prevention and Control of Pneumonia and Diarrhoea – GAPPD” plan (Wardlaw et al., 2006).

Pneumonia can be categorized into hospital-acquired pneumonia, community-acquired mainly affects immunocompromised patients such as aspiration pneumonia or jirovecii pneumonia occur due to large accumulation of gastric or upper airway secretions into the lungs with typical symptoms of cough, hypoxemia, and chest pain (Catherinot et al., 2010, Kim et al., 2018). Therefore, it is imperative to diagnose pneumonia at its early stages to contain complications. The most common causative agents for pneumonia are *Legionella pneumophila*, *Streptococcus pneumonia*, and respiratory viruses (Leber et al., 2018). It is usually difficult to early and timely diagnose pneumonia in childhood due to microbiological tests' less sensitivity and weak clinical interpretation (Al-Antari et al., 2018). Sputum culture is another diagnostic tool but has a limitation of low yield, viability loss for *Homophiles influenza* type b and *S. pneumonia, and* difficulty in differentiating pathogens and colonizers (Eshwara et al., 2020).

Hence, a chest X-ray is considered a potential diagnostic tool for the detection of pneumonia in pediatrics. To further improve the accuracy and quality of diagnosis through chest x-rays and save physician time involved in review and diagnosis. It is highly recommended to utilize recent advancements in technology and software through computer-aided diagnosis for early and accurate pneumonia detection (Barhoom and Abu-Naser, 2022).

Computer algorithms named Artificial Intelligence (AI) exhibit cognitive-like features including the capability to learn which has gained significant importance in the medical field through image analysis in radiology, pathology, dermatology, and prediction of phenotypes from genotypes in genomics (Hosny et al., 2018, Zou et al., 2019). The subfield of AI is machine learning (ML) aids in model building by allowing computers to train the algorithm on the input of data either labeled or unlabeled, statistically analyze data, and solve the problem for automated decision making (Al Barsh et al., 2020). A subset of ML is deep learning which uses neural network (NN) algorithms that act like the visual cortex of the human brain such as the Convolutional neural network (CNN) (Anwar et al., 2018). CNN has been successfully implemented in the medical field in the detection of Alzheimer's disease (Liu et al., 2018), segmentation of brain tumors (Kilicarslan et al., 2020), breast cancer (Toğaçar et al., 2020) and malaria (Gourisaria et al., 2020), and tuberculosis detection (Stirenko et al., 2018). The hybrid CNN models have demonstrated improved automatic detection of mass and classification of tumors, and detection of pneumonia through chest segmentation in comparison to CNN models (Rajaraman et al., 2019). However, there is limited literature on the usability and clinical implication of deep learning models in the early detection and treatment of pneumonia. In this systematic review and *meta*-analysis, we have summarized deep learning models used for the early detection of pneumonia in pediatrics and adults, especially of bacterial origin.

## 2 Material and methods

### 2.1 Ethics

The present study followed the guidelines of the Preferred Reporting Items for a Systematic Review and Meta-analysis of Diagnostic Test Accuracy Studies (PRISMA-DTA). There was no requirement for approval of the institutional review board because only anonymized data was collected from the previous literature. The collected data during the research was utilized solely for research. Moreover, the researcher has adhered to the principles of ethical conduct in the analysis, interpretation, and presentation of the data. The researcher has acknowledged all sources of information and data utilized in the research and has followed ethical practices of academic writing.

### 2.2 Search strategy

**Fig. 1:**
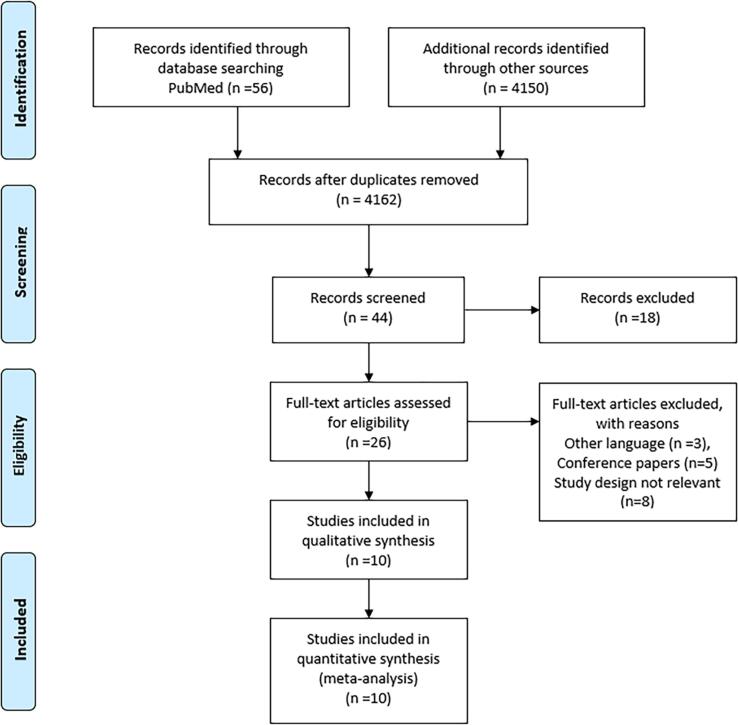
PRISMA diagram to represent screening, eligibility, and inclusion of relevant articles.

This systemic review and *meta*-analysis included a comprehensive literature search for the retrieval of all relevant potential data related to the efficacy of artificial intelligence models in the detection of pneumonia associated with bacterial infection through electronic databases including PubMed, Scopus, and Google Scholar from 1st January 2016 till 1 July 2023. The initial screening of literature showed 56 articles from PubMed, 5 articles from Scopus, and 4153 articles from Google Scholar. Initially, a broad search strategy resulted in the retrieval of 4162 duplicate articles, bibliographies, editorial notes, and non-relevant articles were excluded after initial screening and a total of 47 articles were screened. Further 18 articles were excluded due to only abstracts being available and 29 full-text eligible articles were included. Three articles due to other languages, five conference papers, and eight articles due to not relevant study design were further excluded and the final ten articles were included for qualitative and quantitative analysis. The literature search for this study was conducted following the guidelines of “Preferred Reporting Items for Systematic Review and Meta-Analysis” (Fig. 1) (Moher et al., 2009). The following keywords were used in the literature search strategy ”Efficacy of AI model OR clinical efficacy of AI model AND bacterial pneumonia AND pediatrics, OR artificial intelligence models AND chest x-ray AND pediatrics AND deep learning model”.

### 2.3 Selection criteria

We included studies that reported the detection of pneumonia primarily due to bacterial infection through the use of chest x-ray or blood smear, mainly among the pediatric population, availability of deep learning model efficacy or validity records in terms of accuracy, the area under the curve, sensitivity, specificity, or F measures. The study also included participants from all age groups and both genders. All abstracts, COVID-19-associated pneumonia impact articles, editorials, poster presentations, studies not on human subjects, and articles in languages other than English were excluded. The literature search was done by two independent researchers to access eligibility criteria based on the article's abstract, the full text was further retrieved and studied in detail for relevant articles for comprehensive valuation. The important characteristics of the selected deep learning model including accuracy, the area under the curve, sensitivity, specificity, and F measures were reported in the form of a table to estimate diagnostic values.

### 2.4 Quality assessment

The quality assessment of the included article was done through “STARD 2015” using a checklist of 25 items to check the diagnostic accuracy of included studies to ensure results interpretation through assuring transparency, completeness, and improved research reproducibility (Patrick et al., 2015). The items on the checklist were marked as (yes/no/maybe/ not applicable) by two researchers.

### 2.5 Statistical analysis

The data extracted from the included study articles were maintained on a Microsoft Excel spreadsheet in 2016 for further analysis. Statistical analysis for the *meta*-analysis study was done using Python version 3.11.5 using the packages Pandas, Numpy, Seaborn, and Matplotlib. We calculated pooled sensitivity and pooled specificity and a 95 % confidence interval was calculated as accuracy test measures. The heterogeneity of data was measured to estimate the non-threshold effect using the formula:

I^2^ = max {100 %*[Q-(K-1)]/Q, 0]

## 3 Results

This systematic review and *meta*-analysis were conducted to estimate the predictive accuracy and efficiency of artificial intelligence-based deep learning networks and algorithms in the early detection of pneumonia. A total of 13 articles (Swaminathan et al., 2016, Smith et al., 2018, Correa et al., 2018, Kermany et al., 2018, Hwang et al., 2019, Wu et al., 2020, Hashmi et al., 2021, Hsu et al., 2022, Ortiz-Toro et al., 2022; Ukwuoma et al., 2022; Ali, 2023, Meena et al., 2023, Rajput et al., 2023) were included in the *meta*-analysis. Twelve studies examined the role of deep learning models in the early detection of pneumonia and one study predicted target drug concentrations for the treatment of tuberculosis. One study used a blood smear for clinical laboratory-based diagnosis of bloodstream infection due to gram-positive and gram-negative bacteria and nine studies used chest X-rays to detect pneumonia infiltrates. Eight studies (60 %) studies were conducted among the pediatric population, one study (10 %) among both pediatric and adults, and four studies (30 %) were among adult patients with pneumonia. Overall studies included a total of 126,610 images and 1706 patients in this *meta*-analysis. Convolutional neural networks (Smith et al., 2018, Wu et al., 2020; Hashmi et al., 202) and deep learning neural networks (Kermany et al., 2018, Hwang et al., 2019) were frequently used models, other used AI models included CART (Swaminathan et al., 2016), ANN (Correa et al., 2018), SVM (Hashmi et al., 2021, Ortiz-Toro et al., 2022), and Hybrid Transformer Encoder (Ukwuoma et al., 2022), Visual Geometry Group 19 (Ali, 2023), Long Short-Term Memory (Meena et al., 2023) Bio-Inspired Optimization Based LSTM (Rajput et al., 2023).

### 3.1 Characteristics extracted from included studies

The characteristics extracted from the included studies are mentioned in Table 1. Smith et al. (2018) used a deep convolutional neural network for the interpretation of bacterial organisms through blood count to classify grain stains through a collection of 25,488 positive culture blood strain images of gram-positive cocci (chains and clusters), gram-negative rods or no cells. The CNN model showed a classification accuracy of 94.90 % and AUC > 0.98. 189 slides with the application of a classification algorithm with no human intervention showed sensitivity (98.4 %, 93.2 %, and 96.3 %) for gram-positive cocci (chains), gram-positive cocci (clusters), and gram-negative rods. the highest specificity was reported for gram-negative rods (98.1 %), followed by (97.2 %) gram-positive cocci (clusters) and (75.0 %) for gram-positive cocci chains. This provided potential ground for the technologist to review smears swiftly due to the presence of classified and prescreened crops to correctly identify bloodstream infections and get appropriate antimicrobial treatment accordingly. Similarly, another study used a hybrid novel CNN model in combination with random forest (RF) to create an “ACNN-RF” model for the precise detection of pneumonia strains on chest X-rays with a dataset of 5863 images (Wu et al., 2020). In a retrospective cohort of pediatrics, the mean detection rate was found 97 % indicative of this model's efficacy. 4265 images were of patients with pneumonia and 1574 were of a normal sample. The input size 64*64*3 showed the highest accuracy of 96.9 % in identifying structural features of pneumonia in the lungs in a short period.

**Table 1:** Characteristics of all included studies including the artificial intelligence model used, diagnostic accuracy, area under the curve, sensitivity, specificity, and F measure Table 1**:** Characteristics of all included studies including the artificial intelligence model used, diagnostic accuracy, area under the curve, sensitivity, specificity and F measure.

Author	Year	Micro organism	Purpose	No of Images/ Sample	Smear used/ organ involve	Population	AI Model used	Accuracy	AUC/ ROC	Sensitivity	Specificity	F measure
Smith et al. [37]	2018	Gram-positive cocci in clusters and chains, gram negative rods	Automation of blood culture gram staining	25,488 images	Blood smear	Adult and paediatric	Deep convolutional neural network	94.90 %	>0.98	Gram positive cocci (pairs and chains): 98.4 %, For gram positive cocci (Clusters): 93.2 %, For gram negative rods: 96.3 %	Gram positive cocci (pairs and chains): 75 %, For gram positive cocci (Clusters): 97.2 %, For gram negative rods: 98.1 %	
Wu et al. [46]	2020	Bacterial or viral	Prediction of pneumonia on chest x-ray	5863 images	lungs	Paediatric	ConvolutionalNeural Network - Random Forest (ACNN-RF)	96.90 %		95 %	95.90 %	97.70 %
Swaminathan et al. [40]	2016	M tuberculosis	Target drug concentration predicted for tuberculosis treatment in relation to pneumonia	161 patients	lungs	Paediatric	CART, random forest			0.52–0.86	0.68–0.89	
Correa et al. [9]	2018	Bacterial or viral	Identification of pneumonia infiltrates	60 frames	Skin and subcutaneous tissue in lungs	Paediatric	Artificial neural network (ANN)			>91.52 %	100 %	
Kermany et al. [19]	2018	Bacterial or viral	Prediction of pneumonia on chest x-ray	207 images	lungs	Paediatrics	Neural network (NN) algorithm	92.80 %	96.80 %	93.20 %	90 %	
Hwang et al. [16]	2019	Bacterial or viral	Prediction of pneumonia on chest x-ray	54,221 images	lungs	Adults	deep-learning NN algorithm		0.965 (95 % CI, 0.955–0.975)	95.10 %	75 %	
Ortiz-Toro et al. [30]	2022	Bacterial or viral	Automatic pneumonia detection on chest x ray	5856 images	lungs	Paediatrics	Support Vector Machine, K Nearest Neighbours, Random Forest	Dataset 1: 83.3 % (radiomics), 89.9 % fractal dimension, 91.3 % (superpixel based histon) Dataset 2: 95.3 % (radiomics), 99 % (fractal dimension), 99 % (superpixel based histon	AUC Radiomics (0.919), 0.923 for KNN and fractal dimension, 0.943 for KNN and histon	Dataset 1: 89 % (radiomics), 93.6 % fractal dimension, 90.5 % (superpixel based histon) Dataset 2: 99.2 % (radiomics), 100 % (fractal dimension), 98.6 % (superpixel based histon		
Ukwuoma et al. [43]	2022	Bacterial or viral	Automatic pneumonia detection on chest x ray	15,000 images	lungs	Paediatric	Hybrid deep learning model (Transformer Encoder, TE)	overall 99.21 % (Hybrid TE with ensemble A backbone), 98.28 % (Hybrid TE with ensemble B backbone)	overall 99.18 % (Hybrid TE with ensemble A backbone), 98.28 % (Hybrid TE with ensemble B backbone)	overall 99.19 % (Hybrid TE with ensemble A backbone), 98.28 % (Hybrid TE with ensemble B backbone)	overall 99.20 % (Hybrid TE with ensemble A backbone), 98.28 % (Hybrid TE with ensemble B backbone)	overall 99.21 % (Hybrid TE with ensemble A backbone), 98.28 % (Hybrid TE with ensemble B backbone)
Hashmi et al. [13]	2021	Bacterial or viral	Automatic pneumonia detection on chest x ray	5836 images	lungs	Paediatric	ResNet50 and Compound scaled (CS)	ResNet50: 97 %, CS: 98 %		ResNet50: 98 %, CS: 98 %		ResNet50: 99.50 %, CS:99.71 %
Hsu et al. [15]	2022	Bacterial or viral	Automatic pneumonia detection in high risk patients	1545 patients	lungs	adults	Support Vector Machine (SVM) supported integrated genetic algorithm (GA) named (IGS)	70.11 %	0.7758	73.46 %	69 %	
Ali [3]	2023	Bacterial or viral	Automatic pneumonia detection on chest x ray	5863 images	lungs	Paediatric	VGG-19	94.8 %		96.5 %	94.2 %	
Meena et al. [27]	2023	Bacterial or viral	Automatic pneumonia detection on lungs CT scan	3000 images	lungs	adults	LSTM	99.2 %				98.9 %
Rajput et al. [34]	2023	Bacterial or viral	Automatic pneumonia detection on chest x ray	5216 images	lungs	adults	BIO-LSTM	95 %				97 %

Another study by Swaminathan et al. (2016) examined the drug threshold levels to predict the severity of tuberculosis in pediatrics caused by *Mycobacterium Tuberculosis* through the use of CART and a random forest model to estimate target drug therapeutic concentrations for treatment. Known outcomes were reported in 143 children with 77 % completing treatment with first-line anti-tuberculosis drugs including rifampicin, isoniazid, and pyrazinamide. The ROC curve for boosted CART and the random forest was found 0.75 and 0.77. Both CART and the Random forest model identified pyrazinamide peak concentration (<38.10 mg/L) as the main predictor of treatment failure, which led to the development of new therapeutic target dose concentrations as an important biomarker associated with failure of therapy and mortality among pediatrics with tuberculosis. Characteristic vectors were used from ultrasound images in an artificial neural network to predict pneumonia infiltrates in young children with sensitivity (90.9 %) and specificity (100 %) (Correa et al., 2018).

The model by Kermany et al. (2018) showed an accuracy of 92.80 %, an area under the curve of 96.80 %, a sensitivity of 93.20 %, and a specificity of 90 %. The study showed an improved diagnosis of diabetic macular edema and an improved diagnosis of regions diagnosed through the neural network to improve therapeutic outcomes. The deep-learning NN algorithm presented by Hwang et al. (2019) showed an accuracy of 92.80 %, an area under the curve of 96.80 %, a sensitivity of 93.20 %, and a specificity of 90 %. The study showed an improved diagnosis of diabetic macular edema and an improved diagnosis of regions diagnosed through the neural network to improve therapeutic outcomes. Ortiz-Toro et al. (2022) compared three AI techniques of Support Vector Machine, K Nearest Neighbors, and Random Forest among two data sets of patients to detect pneumonia. The pediatric dataset showed an accuracy of 83.3 % (radiomics), 89.9 % fractal dimension, 91.3 % (superpixel-based histon), and dataset two showed an accuracy of 95.3 % (radiomics), 99 % (fractal dimension) and 99 % (superpixel based histon). The sensitivity of dataset 1 was found 89 % (radiomics), 93.6 % fractal dimension, 90.5 % (superpixel-based histon), and dataset 2: 99.2 % (radiomics), 100 % (fractal dimension), 98.6 % (superpixel-based histon respectively suggestive of reliability as an automatic detection tool for pneumonia.

Ukwuoma et al. (2022) in their study used individual or trained deep learning models transformer encoders including “multi-head self-attention network” and “MLP block for better, generalized, and accurate identification” in comparison to other deep learning models such as Xception, DenseNet201, GoogleNet, InceptResNetV2, VGG16 and EfficientNetB7 to identify pneumonia framework to prevent acute inflammatory responses through the use of ensemble transform strategy. The results showed improvement in binary (2.05 %) and multiclass (1.3 %) through the XAI framework. The hybrid transformer encoder model showed overall high accuracy (99.21 %) with ensemble A, high AUC (99.18 %), high sensitivity (99.19 %), high specificity (99.20 %), and high F score (99.21 %) with ensemble A in comparison to ensemble B backbone, indicative of high success identification of early pneumonia signs on chest x-ray. Therefore, overall the hybrid XAI model in terms of heat maps and saliency maps showed better identification results. Hashmi et al. (2021) examined the detection of pneumonia strains on chest X-rays using a deep learning model named compound scaled and compared it with ResNet50 to improve clinical decision-making by radiologists. The results showed improved accuracy (98.14 %) and area under the curve (AUC) (99.71 %) for the compound scaled model. The F1 score for CS was improved (99.71 %) in comparison to ResNet50 (99.50 %). This computer-aided pneumonia diagnosis can aid in the development of the localized affected area of lung identification.

A study by Hsu et al. (2022) used an AI model based on a Support Vector Machine (SVM) supported integrated genetic algorithm (GA) named (IGS) for the automatic detection of pneumonia in high-risk patients with readmission within thirty days of previous discharge. The IGS model was compared with the deep neural network (DNN) and logistic regression (LR) model and results showed significantly improved accuracy of the IGS model (70.11 %) in comparison to the LR model (65.77 %) and DNN model (61.50 %). The IGS model utilized OB2 objective functionality. The sensitivity, area under ROC curve - AUC, and specificity of the IGS model were (73.46 %, 0.7758, and 69.26 %) respectively, in comparison to the DNN model (79.34 %, 0.7547, and 56.95 %) and LR model (78.44 %, 0.7689, and 62.54 %). This IGS model provided the base for further evaluation to improve treatment outcomes through the introduction of suitable timely interventions among high-risk patients to avoid readmission, disease and symptoms severity, and cost of healthcare procedures. Patients with congestive heart failure showed more readmission with Mean ± SD 2.5 ± 7.8. The study also used forty-nine variables with a significant difference reported among 34 variables (p < 0.05).

A study by Ali (2023) performed a retrospective cohort study by utilizing an AI model based on the VGG-19 model for detection of pneumonia. This deep learning model was compared with Transfer Learned InceptionV3 and VGG-19 outperformed the other model. Data of pediatrics were taken from age 1 to 5 years and a total of 5863 CXR images datasets were taken. The model showed 94.8 % accuracy, 96.5 % sensitivity and 94.2 % specificity. This model showed a base for further evaluation to enhance treatment results through the introduction of suitable interventions among high-risk patients.

Another study by Meena et al. (2023) employs the LSTM model to predict pneumonia. The study utilized 3000 images of CT scans of the lungs. The model reported 99.2 % accuracy, 99.2 % specificity, 99.3 % sensitivity and 98.9 % F1-score. The model outperformed the GRU model, and it was concluded that the LSTM model generate excellent outcomes for predicting negative and positive cases for pneumonia.

Rajput et al. (2023) proposed a novel model bio-inspired optimization based on Long Short-Term Memory (BIO-LSTM), for the prediction of pneumonia using 5216 (3875 infected patients and 1341 normal) CXR images datasets pediatrics. The model outperformed other conventional approaches by depicting an accuracy of 95 %, precision of 94 %, sensitivity of 96 %, specificity of 95 % and F1 score of 97 %. it was concluded that it would assist physicians in detecting patients more accurately and quickly enhancing patient results.

[Insert Table 1 here].

### 3.2 Pooled estimation of sensitivity and specificity

**Fig. 2:**
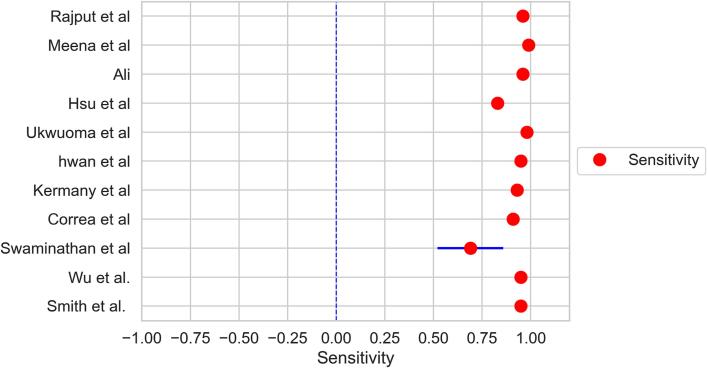
Forest plot indicating the pooled sensitivity of deep learning model in estimating diagnostic accuracy to detect pneumonia.

**Fig. 3:**
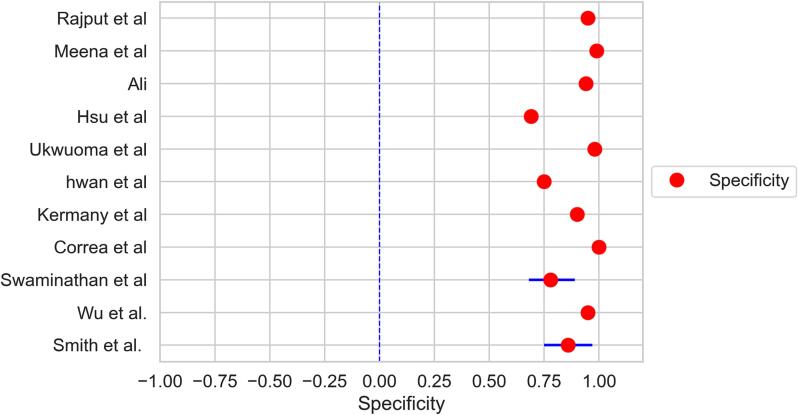
Forest plot indicating the pooled specificity of deep learning model in estimating diagnostic accuracy to detect pneumonia.

The forest plots demonstrated in Fig. 2, Fig. 3 were constructed for pooled estimates of the sensitivity and specificity of deep learning models' diagnostic accuracy in the identification of pneumonia strains through chest X-rays. Each red dot represents an individual study and represents the pooled sensitivity (Fig. 2) and pooled specificity (Fig. 3) across all the studies. At a 95 % confidence interval, pooled sensitivity was 0.90 (0.85–0.94), and I2 statistics were 90.20 (88.56 – 91.92). Whereas, the pooled specificity for deep learning models' diagnostic accuracy was 0.89 (0.86–0.92) and I2 statistics 92.72 (91.50 – 94.83). The forest plot for the sensitivity and specificity of AI-used models from different studies showed that almost all the studies do not cross the line of no effect and the results for the sensitivity of AI model used were significant. This analysis reveals remarkably low heterogeneity, signifying a high degree of consistency across all the included studies. This is a particularly positive finding, as it highlights the robustness and reliability of the pooled sensitivity and specificity estimates. Researchers and healthcare professionals can have confidence in these estimates, knowing that they accurately represent the consistent performance of deep learning models in detecting pneumonia across various clinical settings.

## 4 Discussion

This study deals with the early detection of Pneumonia with the help of deep-learning models. There is no specific experimentation but this research has been supported by a literature-based systematic review and *meta*-analysis. The implication of AI has been considered to have the nature of the black box. This natural technique has been very helpful in activating the map of visualization and developing the interest of attention. The models of deep learning explain the possibility of determining the efficiency of possibilities. As one the study indicated, the systematic review successfully adopted the map feature with the help AI algorithm for the development and future establishment of useful work of endoscopists. The main targeted detection was a biopsy for *H pylori infection* (Nakashima et al., 2018). In the studies of IEEE, the main goal of techniques has been proven very beneficial on the level of replacing the histologic examination with the help of mucosal features. It has represented the accuracy in the diagnosis of infection. This has to reduce the time of detection and increase the examination of the histological process (Takeda et al., 2020). The application of AI can be further evaluated for the collection of major data regarding the required research.

With the rising emergence of communicable and infectious diseases in terms of viral flues, pneumonia is a serious public health concern. Various contributing factors including dense populations, environmental, demographic, and behavioral patterns, and hygiene conditions contribute to the reproduction of pathogens (Jain, 2020). The development of deep learning neural algorithms significantly improved risk assessment in immunocompromised patients and improved clinical outcomes. This *meta*-analysis identified pneumonia as the important cause of pediatric and adolescent-associated morbidity and mortality and summarized the recent use of deep learning models to improve the early detection of bacterial pneumonia and infections in pediatrics and adults. Our study highlighted the use of combination models including ACNN-RF, CART, Hybrid Transformer Encoder (AUC: 99.18 %), and integrated genetic algorithm (IGS) (AUC: 0.7758) for the early detection of pneumonia to avoid complications.

Another study conducted in 20 countries tested and validated a novel tool “PREPARE” for the early risk assessment of pneumonia in pediatrics aged between 2 and 59 months to reduce cyanosis, convulsion, hypoxemia, and hospital-associated deaths (AUC: 0.83) (Rees et al., 2022). The effect of AI models on chest imaging for diagnostic accuracy has been identified in one of the systemic reviews and *meta*-analyses. Chest imaging distinguishes COVID-19 patients from cases of pneumonia. The models of QUADAS-2, the CLAIM checklist, and the tool of RQS have been used to interpret the clinical application and further research work has been required. This study includes many independent surveys to acquire favorable pools of results to identify the sensitivity with their specificity and AUC. The results of the study have been significant but unable to determine clear effective images due to poor quality of methodological practices. However, the diagnosis of pulmonologists shows that the study has positive responses toward the diagnostic accuracy of AI models. It has a high potential analysis toward the distinguishing characteristics of COVID-19 patients from cases of pneumonia. Moreover, this positive implication has been highlighted in the present studies (Jia et al., 2022).

Many studies have not given an explanation of AI algorithms and complete information in the database. About only 6 % of studies use the code language for the study models which readers can access the open and the closed data to robust their research. Therefore, analysis of different groups and subgroups identifies five key factors for developing the modalities of different imaging which have been based on X-ray diagnosis. The study concludes that AI models have been more effective and better than CT-based models. They have been not more convincing as compared to deep learning models. The pooled result of both models varies and indicates that DL models are to be more valuable than ML (Kumar et al., 2012). The main advantage of deep learning is that it does not require any kind of manual learning or extraction. There is no use for manpower and extra effort. It provides an artificially designed sophisticated system for radiomic analysis. Mostly it has been observed, that the most frequent and common model which have been used in the research is the DL model. This model is highly inspired by the Convolutional Neural Network which regulates the biological and visual mechanism of the visual rectified layer and fully connected layer (Shin et al., 2016).

However, the present study reported low heterogeneity among contexts and ununiformed reporting of pneumonia-associated morbidity and mortality. Our study results showed a total 95 % confidence interval for included studies pooled sensitivity was 0.90 (0.85–0.94) and pooled specificity for deep learning models' diagnostic accuracy was 0.89 (0.86–0.92). Another study used the Xception model for the detection of pneumonia in comparison to the Vgg16 model and showed improved sensitivity (0.85 %) normal pneumonia recall (0.94 %) and precision (0.86 %) (Ayan and Ünver, 2019).

According to a recent study, antimicrobial resistance has always faced the core ground challenges to modern health care. The clinical implementation and the possibilities of procedures for diagnostic laboratories cannot be completed without the help of AI algorithms and DL methods. Quality of data with accuracy has been established by its practical implication. The control of viral diseases has been monetized to meet International standards. The genome sequencing of the microbes in clinical laboratories with quality system management robust the efficiency of the system. The NLP-based technique also has been used to extract unstructured data which has been recommended as more cost-effective and accurate for maintaining the electronic medical and health records (Forde et al., 2022).

Another study used the CART model among schizophrenic patients for the detection of pneumonia in addition to the other six employed methods, the accuracy of CART was reported at 0.804, and the AUC value > 0.8 indicative of the good performance of the model (Kuo et al., 2019). A CNN 22-layer model was designed using a support vector machine, K-nearest neighbor, and random forest classifier and used on 5856 chest x-rays to identify pneumonia infiltrates with a diagnostic accuracy of 99.52 % and AUC (98.7 %) (Sourab and Kabir, 2022). However, the hybrid model mentioned in our *meta*-analysis used had an accuracy of 99.21 % and AUC of 99.18 % in ensemble A and an accuracy of 98.285 and AUC of 98.28 % in ensemble B. Another study used a deep hybrid learning model for pneumonia detection logistic regression and SVM with radial basis function and showed an accuracy of 98.55 % (Yaseliani et al., 2022).

Our study has a limitation of limited generalizability of findings and further identification of potential risk factors is required. However, there is limited literature on the usability and clinical implication of deep learning models in the early detection and treatment of pneumonia. One of the study limitations is the population which meets the validity of the research. The performance of diagnosis depends on the targeted population and the prevalence of the selected population. This study applies different models of implication in specific validation of datasets. This is also the limitation of this study. The study provides a limitation to generalizing the predictive models which can be affected by human error and cause various segmentation. Especially in the mechanism of microorganism detection most AI techniques, research tools, and software insufficient data replicate the data. This has to be controlled efficiently to increase routine reproducibility in clinical applications.

## 5 Conclusion

This explanatory systematic review and *meta*-analysis highlighted the recent deep learning models single or in combination with high accuracy, sensitivity, and specificity to ensure reliable use for bacterial pneumonia identification and differentiate from other viral, and fungal pneumonia in children and adults through chest x-rays and radiographs. This will help in the prompt selection of appropriate and precise medical and therapeutic protocol and the detection of safe therapeutic doses of medicines to treat pneumonia and associated comorbidities. Furthermore, The model of deep learning is susceptible to overfitting, which happens when the model learns data training too well and as a result, it is unable to recognize the new data. Moreover, most of the time deep learning model interpretation is difficult which makes it challenging to comprehend the data and make decisions on the basis of outcomes.

Data availability

The authors declare that all the data included in this study are available within the paper and its supplementary information files.

## Declaration of competing interest

The authors declare that they have no known competing financial interests or personal relationships that could have appeared to influence the work reported in this paper.
